# Effects of challenge dose and inoculation route of the virulent *Neospora caninum* Nc-Spain7 isolate in pregnant cattle at mid-gestation

**DOI:** 10.1186/s13567-019-0686-3

**Published:** 2019-09-23

**Authors:** Patricia Vázquez, Koldo Osoro, Miguel Fernández, Alicia Román-Trufero, Javier Regidor-Cerrillo, Laura Jiménez-Pelayo, Marta García-Sánchez, Silvia Rojo-Montejo, Julio Benavides, Pilar Horcajo, Luis Miguel Ortega-Mora

**Affiliations:** 1 SALUVET, Animal Health Department, Faculty of Veterinary Sciences, Complutense University of Madrid, Ciudad Universitaria s/n, 28040 Madrid, Spain; 2 Regional Service for Research and Agri-Food Development (SERIDA), 33300 Villaviciosa, Asturias Spain; 3 Mountain Livestock Institute, Animal Health Department, University of León CSIC-ULE, 24346 Grulleros, León Spain; 4 SALUVET-Innova S.L., Faculty of Veterinary Sciences, Complutense University of Madrid, Ciudad Universitaria s/n, 28040 Madrid, Spain

## Abstract

Parameters such as pathogen dose and inoculation route are paramount in animal models when studying disease pathogenesis. Here, clinical findings, including foetal mortality, parasite transmission rates and lesion severity, and immune responses were evaluated in Asturiana pregnant heifers at day 110 of gestation challenged with a virulent (Nc-Spain7) *Neospora caninum* isolate. Four different doses of parasite tachyzoites were inoculated intravenously (IV1, 10^7^ parasites, *n* = 6; IV2, 10^5^, *n* = 6; IV3, 10^3^, *n* = 6; and IV4, 10^2^, *n* = 5), and the subcutaneous (SC) inoculation route was also assessed for the dose of 10^5^ tachyzoites (SC, *n* = 6). In addition, a control group (*n* = 4 pregnant heifers) was evaluated. Foetal death was observed in all infected groups from 25 to 62 days post-infection, varying with the dose (IV1:4/6, IV2:3/6; IV4:2/5, IV3:1/6), and was three times less frequently associated with the SC route than IV inoculation (1/6 vs. 3/6). A dose-dependent effect for parasite loads in placental and foetal brain tissues was also detected. After SC challenge, a reduced number of tachyzoites were able to reach foetal brain tissues, and no lesions were observed. In calves, specific IgG responses in precolostral sera were mainly associated with high-dose groups (IV1 [100.0%] and IV2 [66.7%]), and cerebral parasite DNA detection was scarce (3/18). In dams, IFN-γ production and the dynamics of anti-*N. caninum* IgG antibodies varied with the dose, and the cell-mediated immune response was also found to be route-dependent. Our results confirm the influence of parasite dose and inoculation route on the outcome and dynamics of bovine neosporosis at mid-gestation.

## Introduction

Infection with *Neospora caninum* is widely recognized as one of the major causes of bovine abortion worldwide (reviewed by [1, 2]) and it is responsible for large economic losses, particularly for the dairy industry, according to an economic study considering ten countries, including the worldwide leading cattle industries [3].

In naturally infected cattle herds, the spread of this apicomplexan parasite is mainly associated with efficient transplacental infections following reactivation of a chronic infection during gestation in the dam [4, 5], although horizontal transmission through oocyst ingestion is also possible and more frequent than previously thought [6, 7]. The clinical outcome of *N. caninum* infection can be abortion during months 3–9 of gestation (mostly between 5 and 6 months of gestation), a still-born calf, a new-born calf with neurological clinical signs, or a clinically healthy but persistently infected newborn calf [1, 2].

Although the disease pathogenesis remains incompletely understood, clinical features of neosporosis in pregnant cattle appear to be influenced by the stage of gestation at infection, being less severe as gestation progresses. Hence, and as demonstrated by experimental bovine challenge models, within the first term of gestation, foetal death is likely to occur after inoculation of NC1 [8–11] and Nc-Liverpool isolates [12–14], or (more recently) using well-characterized virulent isolates, such as Nc-Spain7 [15, 16]. In contrast, experimental infections during the second and third term of gestation have mostly led to the birth of persistently infected calves [12–14]. However, some *N. caninum* experimental primary infections in naïve dams at 110 days of pregnancy can also result in foetal death [17, 18].

Pregnant models at mid-gestation are models that better mimic the dynamics and outcome of natural infections [19], making them especially interesting. As has been widely reviewed by [20], several bovine models have been developed with large variations in describing clinical outcomes between studies. These, among others, could be related to the route of inoculation [i.e., intravenous (IV), intramuscular or subcutaneous (SC)], as well as to the parasite isolate [20]. In particular, these findings highlight that the isolates used previously were not as well characterized and controlled as Nc-Spain7 and that their behaviour can be affected by their in vitro passage [20].

In recent years, the dose-dependent effects of virulent Nc-Spain7 isolate infection have been evaluated in pregnant mouse and ovine models [21, 22]. In dairy cattle, foetal death was reported in 3 of 6 experimentally Nc-Spain7-infected naïve Friesian heifers at mid-gestation during the studied period (up to 7 weeks post-infection) [18]. However, a titration dose of this isolate has not yet been done in bovine models. In addition, it is known that the route of inoculation can result in different effects in pregnant cattle [11] and sheep [22] at mid-gestation.

The aim of this study was to investigate the effect of four challenge doses of a well-characterized, virulent, low-passage *N. caninum* isolate (Nc-Spain7) intravenously inoculated, as well as to determine the effect of the route of inoculation for one of such challenge doses. Our findings provide descriptions of clinical outcome (foetal mortality), parasite distribution and burden, lesion development in placental and foetal/calf tissues, and immune response trends in both dams and foetuses/calves.

## Materials and methods

### Animals and experimental groups

Thirty-three Asturiana breed heifers, aged 23.8 months on average, were housed in the cattle facilities belonging to the Regional Service for Agri-food Research and Development (SERIDA) (Villaviciosa, Spain). Seronegative status for *N. caninum* infection was confirmed by an in-house indirect enzyme-linked immunosorbent assay (ELISA) using soluble antigen [23]. Additionally, the sanitary status of the heifers included screening for the absence of specific antibodies against three other main infectious diseases affecting cattle: bovine viral diarrhoea (BVD) virus, infectious bovine rhinotracheitis (IBR) virus, and *Mycobacterium avium* subsp. *paratuberculosis.* Other details regarding both the health and reproductive management of heifers are provided in Additional file 1.

Pregnant heifers were randomly assigned to five experimentally infected groups (IV1, IV2, IV3, IV4, and SC) and an uninfected control group (Control). At 110 days of gestation (dg), 23 heifers were inoculated via the IV route into the jugular vein with decreasing doses from the virulent Nc-Spain7 isolate (IV1: 10^7^ tachyzoites, *n* = 6; IV2: 10^5^ tachyzoites, *n* = 6; IV3: 10^3^ tachyzoites, *n* = 6; IV4: 10^2^ tachyzoites, *n* = 5), and six heifers were subcutaneously challenged over the left sub-iliac lymph node with 10^5^ tachyzoites of the same isolate (SC) (Table 1). The remaining four uninfected pregnant heifers (Control) received a 2 mL inoculum of phosphate-buffered saline (PBS) by the IV route on day 110 of pregnancy (Table 1).

**Table 1:** **Clinical outcome, serology, histopathology, and parasite detection in tissues for Nc-Spain7 infected foetuses and calves**

Group (dose, route)	Animal ID	Foetal death (dpi)/calf	*N. caninum*-specific IgG		Histopathology (HE, IHQ)	*N. caninum* DNA detection		
			IFAT titre^b^	RIPC		Placentome	Brain	Liver
IV1 (10^7^ tachyzoites)	IV1.1	Foetal death (31)	−	−	+	9/9	3/3	0/3
	IV1.2	Foetal death (25)	−	−	+	9/9	3/3	0/3
	IV1.3	Foetal death (27)	ND	−	ND	ND	ND	ND
	IV1.4	Calf	3200	14.4	−	−	0/10	0/3
	IV1.5	Calf	800	7.8	−	−	2/10	0/3
	IV1.6	Foetal death (30)	−	−	+	9/9	3/3	0/3
IV2 (10^5^ tachyzoites)	IV2.1	Foetal death (29)	ND	−	+	8/8	3/3	1/3
	IV2.2	Foetal death (39)	−	−	+	9/9	3/3	0/3
	IV2.3	Calf	3200	48.5	−	−	0/10	0/3
	IV2.4	Calf	−	− 2.5	−	−	0/10	0/3
	IV2.5	Foetal death (39)	16	−	+	7/9	3/3	0/3
	IV2.6	Calf	6400	60.5	−	−	0/10	0/3
SC (10^5^ tachyzoites)	SC.1	Calf	−	− 4.4	−	−	0/10	0/3
	SC.2	Foetal death (26)	−	−	−	9/9	3/3	1/3
	SC.3	Calf	−	− 4.4	−	−	0/10	0/3
	SC.4	Calf	−	− 4.1	−	−	0/10	0/3
	SC.5	Calf	−	− 3.5	−	−	0/10	0/3
	SC.6	Calf	−	− 4.5	−	−	0/10	0/3
IV3 (10^3^ tachyzoites)	IV3.1	Calf	−	− 2.9	−	−	0/10	0/3
	IV3.2	Calf	−	− 3.1	−	−	0/10	0/3
	IV3.3	Foetal death (38)	ND	−	+	8/9	0/3	ND
	IV3.4	Calf	−	− 3.2	−	−	0/10	0/3
	IV3.5	Calf	50	− 3.3	−	−	0/10	0/3
	IV3.6	Calf	−	− 3.1	−	−	0/10	0/3
IV4 (10^2^ tachyzoites)	IV4.1	Foetal death (62)	32	−	+	8/9	0/3	0/3
	IV4.2	Foetal death (42)	−	−	+	8/9	3/3	0/3
	IV4.3	Calf	−	− 2.9	−	−	0/10	0/3
	IV4.4	Calf	−	− 3.2	−	−	4/10	0/3
	IV4.5	Calf	−	− 3.7	−	−	4/10	0/3

ID: identification, ND: no data are available, IV: intravenous, SC: subcutaneous, HE: haematoxylin–eosin, IHQ: immunohistochemical labelling.

### Culture-derived tachyzoites of the Nc-Spain7 isolate, dose preparations, and administration

Maintenance of *N. caninum* tachyzoites of the Nc-Spain7 isolate in a monolayer culture of MARC-145 cells [24], and subsequent preparation of inocula were performed as previously described [25]. Briefly, tachyzoites (passage 10) were recovered from MARC-145 culture flasks when they were still largely intracellular (at least 80% of undisrupted parasitophorous vacuoles), and cells were repeatedly passed through a 25-gauge needle at 4 °C. The viable tachyzoite number was determined by Trypan blue exclusion followed by counting in a Neubauer chamber (typically between 95 and 99%). Next, tachyzoites were resuspended in PBS and adjusted to the required doses (10^7^, 10^5^, 10^3^, and 10^2^) in a final volume of 2 mL. Challenge doses were administered within 60 min of harvesting from cell culture.

### Clinical monitoring and sample collection

Pregnant heifers were observed daily throughout the entire experimental period. Foetal viability was assessed by monitoring heartbeat and movements by weekly ultrasound scanning (US) during the first 9 weeks post-infection (wpi). Afterwards, three examinations were performed monthly.

Rectal temperatures were recorded daily within the first 14 days post-infection (dpi), and next weekly temperature records were taken until 9 wpi. Rectal temperatures above 39.5 °C were considered to be a clinical sign of febrile reaction.

In heifers from the SC group, changes in the left sub-iliac lymph node after SC challenge were evaluated by palpation and compared to the right sub-iliac lymph node, daily from 0 to 14 dpi and then weekly (until 9 wpi). To characterize these changes, a score was established based on the following criteria: (0) no changes, (1) an enlarged lymph node, (2) pain on palpation, and (3) both an enlarged lymph node and pain on palpation.

Blood samples from pregnant heifers were collected by coccygeal vein puncture into lithium heparin- and silicone-coated Vacutainer tubes (Becton–Dickinson and Company, Plymouth, UK) for the lymphoproliferation assays and to obtain serum samples, respectively. The time schedule for the 14 blood samplings was the following: 0, 1 wpi (4 and 7 dpi), weekly from the 2nd to the 9th wpi, and monthly until the 22th wpi. Serum samples and supernatants from the lymphoproliferation assays were maintained at −80 °C until laboratory analysis for the evaluation of humoral immune responses.

When foetal death occurred, placental and foetal samples were collected as follows. For each placenta, 9 randomly selected placentomes were sampled and divided for storage in 10% neutral buffered formalin for histopathological evaluation and for freezing (−80 °C) for molecular analysis. Foetal fluids (thoracic and abdominal) were collected when possible and stored at −80 °C for serology. With regard to foetal tissues, skeletal muscle, heart, lung, spleen, thymus, mediastinal lymph node, brain and liver samples were properly collected for histopathological (skeletal muscle, heart, lung, spleen, thymus, brain and liver) and molecular analysis (brain and liver), as mentioned above for placentomes.

When the gestation was not interrupted by foetal death, during the week due for delivery, heifers were closely monitored and newborn calves were sampled for blood at birth to obtain precolostral sera and clinically examined according to the following parameters: respiration, hair coat appearance, peripheral oedema, mucous membranes, response to reflex stimulation, muscle tone, heart rate, rectal temperature, sternal recumbence and attempts to rise and suckle, as previously suggested by [26]. Animals were culled within the first weeks of life (1 to 20 days of life). First, calves were sedated by the intramuscular injection of xylazine (Rompun™, Bayer, Mannhein, Germany) (dose 0.3 mg/kg), and then immediately euthanized by an IV overdose of embutramide and mebezonium iodide (T61, Intervet, Salamanca, Spain). At necropsy, fresh and formalin-fixed samples were collected for *N. caninum* DNA detection by PCR (brain and liver) and histopathology (brain).

### Tissue DNA extraction and *N. caninum* PCR detection and quantification

Methods for DNA extraction from ruminant tissues as well as PCR and qPCR protocols have been previously published in the literature [16, 22, 25]. Automated genomic DNA extraction from 20 to 100 mg of maternal (placentomes), foetal (brain and liver) and calf (brain) tissues was performed with the Maxwell^®^ 16 System (Promega, Wisconsin, USA), using the commercial Maxwell^®^ 16 Mouse Tail DNA Purification Kit, following the manufacturer’s recommendations. All placentomes were processed once; brain and liver tissues were assessed in triplicate for foetuses, and 10 sections of brain from each calf were analysed. Parasite DNA detection was carried out by a nested PCR adapted to a single tube for the amplification of the internal transcribed spacer (ITS1) region of *N. caninum* using external primers (TgNN1–TgNN2) and internal primers (NP1–NP2), as previously described [16, 27, 28]. Samples that tested positive in the PCR were further quantified for parasite DNA using qPCR [29, 30]. More detailed information about molecular protocols is given in Additional file 1.

### Histopathology and immunohistochemistry

Ten percent formalin-fixed tissue samples from foetuses and calves were trimmed, embedded in paraffin wax, and conventionally processed for haematoxylin and eosin (HE) staining and histological evaluation. Immunohistochemical labelling of *N. caninum* antigens was performed in placenta and brain sections, and an in-house anti-*N. caninum* polyclonal serum was used to perform the immunohistochemistry protocol according to [31]. After examination of the histological and immunohistochemical slides, each foetus or calf was classified as showing lesions characteristic of *N. caninum* infection (placenta: multifocal areas of necrosis surrounded by inflammatory cells; brain: multifocal non-suppurative necrotizing encephalitis) [20] or no lesions.

### Peripheral blood stimulation assay and quantification of interferon-gamma (IFN-γ) release

Heparinized blood samples were cultured in duplicate with *N. caninum* soluble extract antigen at 5 μg/mL [23], concanavalin (ConA) (Sigma-Aldrich, Madrid, Spain) at 5 μg/mL as a positive control, and PBS as a negative control. Heparinized blood culture supernatants were collected at 24 h after incubation (temperature: 37 °C, CO_2_ level: 5%, humidity: 100%) to measure bovine IFN-γ concentrations by using a commercial ELISA kit (Mabtech AB, Nacka Strand, Sweden). Further details on these protocols are provided in Additional file 1.

### *Neospora caninum*-specific IgG response: ELISA and IFAT

Specific IgG antibody responses against *N. caninum* in heifers and calves were measured by an in-house indirect ELISA [23]. For each serum sample, the optical density value was converted into a relative index percent (RIPC). *N. caninum*-specific IgG responses in foetal fluids and precolostral sera were detected by indirect fluorescent antibody test (IFAT), as previously described [23]. A more detailed description of both serological techniques is given in Additional file 1.

### Statistical analysis

Occurrence of foetal death was analysed by the Kaplan–Meier survival method. Foetal survival curves were then compared by the Log-rank (Mantel–Cox) test, and differences in foetal death rates between groups were assessed by the χ^2^ or Fisher’s exact test. Rectal temperature values were analysed using the two-way ANOVA of repeated measures test until 4 wpi and one-way ANOVA test afterwards. Differences in *N. caninum* detection by ITS1-PCR were evaluated using the χ^2^ or Fisher’s exact test. Differences in parasite burdens were analysed using the non-parametric Kruskal–Wallis test followed by Dunn’s test for comparisons between experimental groups and control group, and the Mann–Whitney test for pairwise comparison if routes of inoculation were investigated (IV vs. SC, IV vs. Control, and SC vs. Control). Humoral and cellular immune responses in heifers for each experimental group up to 4 wpi were analysed using the two-way ANOVA of repeated measures test, and Tukey’s post-test was applied to examine all possible pairwise comparisons at each sampling time. Afterwards, one-way ANOVA test was used to evaluate the level of IgG antibodies until the end point. Antibody responses in foetuses were compared by using Fisher’s exact test for categorical results (positive vs. negative), the non-parametric Kruskal–Wallis test followed by Dunn’s test for comparisons between experimental groups and the control group, and the Mann–Whitney test for pairwise comparisons when the route effect was evaluated (IV vs. SC, IV vs. Control, and SC vs. Control). Statistical significance for all analyses was established at *P* < 0.05. All statistical analyses were carried out using GraphPad Prism 6.01 software (San Diego, CA, USA).

## Results

### Clinical observations

**Figure 1:**
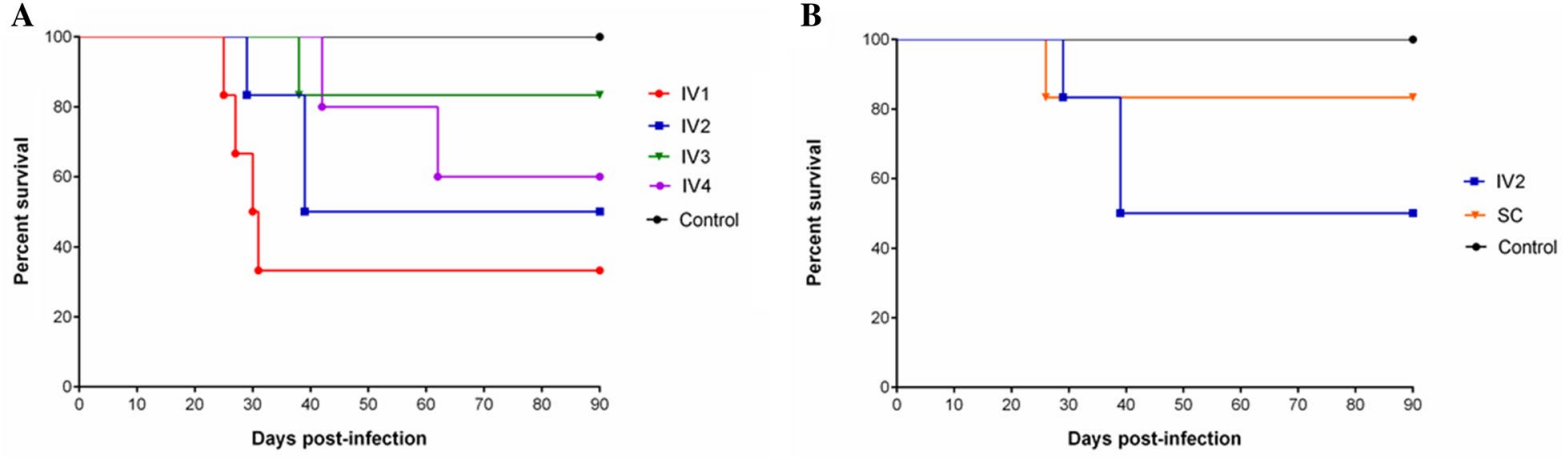
**Effect of challenge dose and route of inoculation of Nc-Spain7 isolate on abortion.** Kaplan–Meier survival curves for foetuses from heifers intravenously challenged with 10^7^ (IV1), 10^5^ (IV2), 10^3^ (IV3), and 10^2^ (IV4) tachyzoites and the uninfected control group (**A**), and intravenously (IV2) and subcutaneously (SC) challenged pregnant heifers with the dose of 10^5^ tachyzoites and the uninfected control group (**B**). Each point represents the percentage of surviving animals at that day and downward steps correspond with observed deaths.

In total, foetal death was detected in eleven heifers. One heifer from the IV1 group (IV1.3) aborted (confirmed by us) on 27 dpi, but neither the placental tissue nor the foetus could be recovered. Foetal death occurred in all experimentally infected groups (IV1: 4/6, IV2: 3/6, SC: 1/6, IV3: 1/6 and IV4: 2/5), between 25 and 62 dpi (Table 1). The median values for foetal survival for the IV1 and IV2 groups were 30.5 and 64.5 days, respectively (Figure 1A). Concerning the challenge dose, there was no statistically significant difference in the comparative analysis of foetal survival curves between experimental groups (*P *> 0.05), or with abortion rates (*P *> 0.05). However, a tendency towards statistical significance of a lower percentage and delayed presentation of abortions and a delayed presentation of them was found when the dose of inoculated tachyzoites was reduced from 10^7^ (IV1) to 10^3^ (IV3) (IV1 > IV3: *P* = 0.0542) (Figure 1A). In terms of inoculation routes, there were no significant differences in the foetal survival rate between IV2 and SC infected groups (*P* > 0.05) (Figure 1B). Healthy calves were delivered from all dams that completed gestation from both infected and control groups.

**Figure 2:**
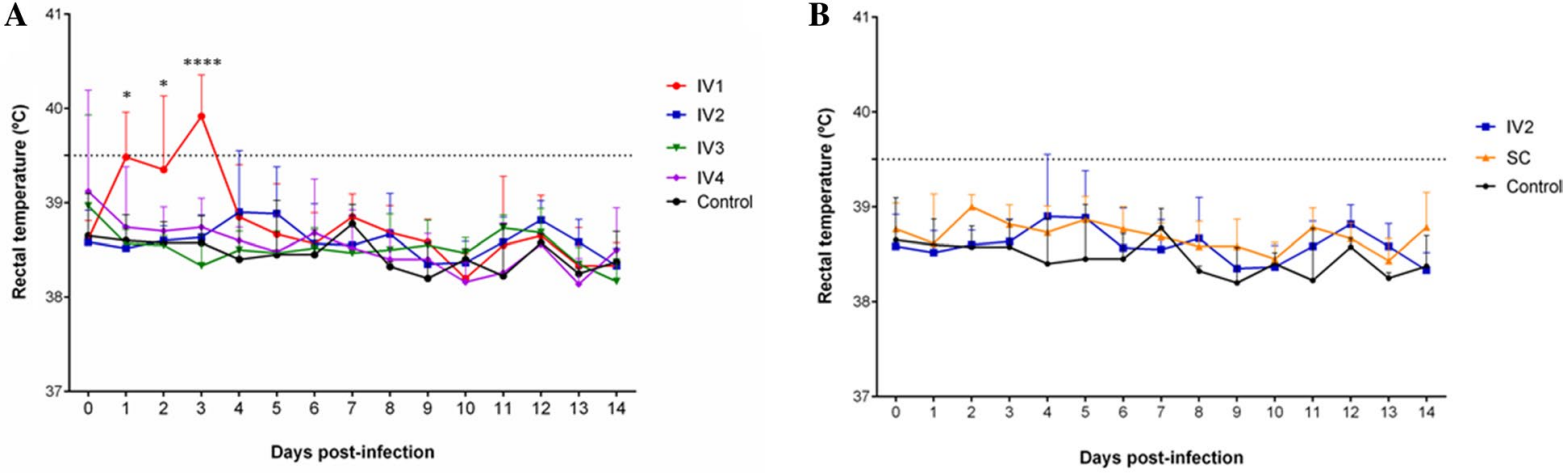
**Rectal temperatures within the first 14** **days post-infection after inoculation of the Nc-Spain7 isolate.** Temperatures of pregnant heifers intravenously challenged with 10^7^ (IV1), 10^5^ (IV2), 10^3^ (IV3), and 10^2^ (IV4) tachyzoites and the uninfected control group (**A**). Temperatures of pregnant heifers intravenously (IV2) and subcutaneously (SC) challenged with the dose of 10^5^ tachyzoites and the uninfected control group (**B**). The spaced line highlights 39.5 °C considered as fever. Each point represents the mean + SD (standard deviation) at different times for each group. Notice a significant increase in mean rectal temperature records in IV1 group compared to IV2, IV3, and IV4 groups within the first 3 dpi. ****P < 0.0001 and *P < 0.05.

All heifers intravenously challenged with the highest dose (IV1) showed febrile reactions (above 39.5 °C) at some time point within the first four dpi (Additional file 2). For this group, a significant increase in mean rectal temperature was found at 1 and 3 dpi compared to pre-infection records (38.6 °C) (1 dpi: 39.5 °C, *P *< 0.01; and 3 dpi: 39.9 °C, *P* < 0.0001) (Figure 2A) and to mean rectal temperature records from the control group (1 dpi: 38.6 °C, *P *< 0.01; 2 dpi: 38.6 °C, *P* < 0.05; and 3 dpi: 38.6 °C, *P* < 0.0001) (Figure 2A). In the other intravenously infected groups (IV2, IV3 and IV4), some individual febrile records were detected for the IV2 and IV4 groups within the first 7 dpi (Additional file 2); however, mean rectal temperatures did not reach 39.5 °C during the monitoring period (up to 9 wpi). An effect of dose on rectal temperature was detected during the period from 1 to 3 dpi based on higher records in the IV1 than in the IV2, IV3 and IV4 groups (1 dpi: IV1 > IV4 > IV3 > IV2, *P* < 0.05; 2 dpi: IV1 > IV4 > IV2 = IV3, *P* < 0.05; 3 dpi: IV1 > IV4 > IV2 > IV3, *P* < 0.0001) (Figure 2A). None of the heifers of the SC group and none of the control heifers developed fever during the monitoring period (up to 9 wpi) (Figure 2B). No significant changes were found in rectal temperatures in the infected groups from 5 to 9 wpi (*P* > 0.05). Changes in the left sub-iliac lymph node were observed in all heifers that were subcutaneously challenged. Lymphadenomegaly (score 1: enlarged lymph node) was observed predominantly between 4 and 14 dpi (83–100% of heifers) but resolved before 29 dpi.

### Parasite DNA distribution and burden in placental, foetal and calf tissues

**Figure 3:**
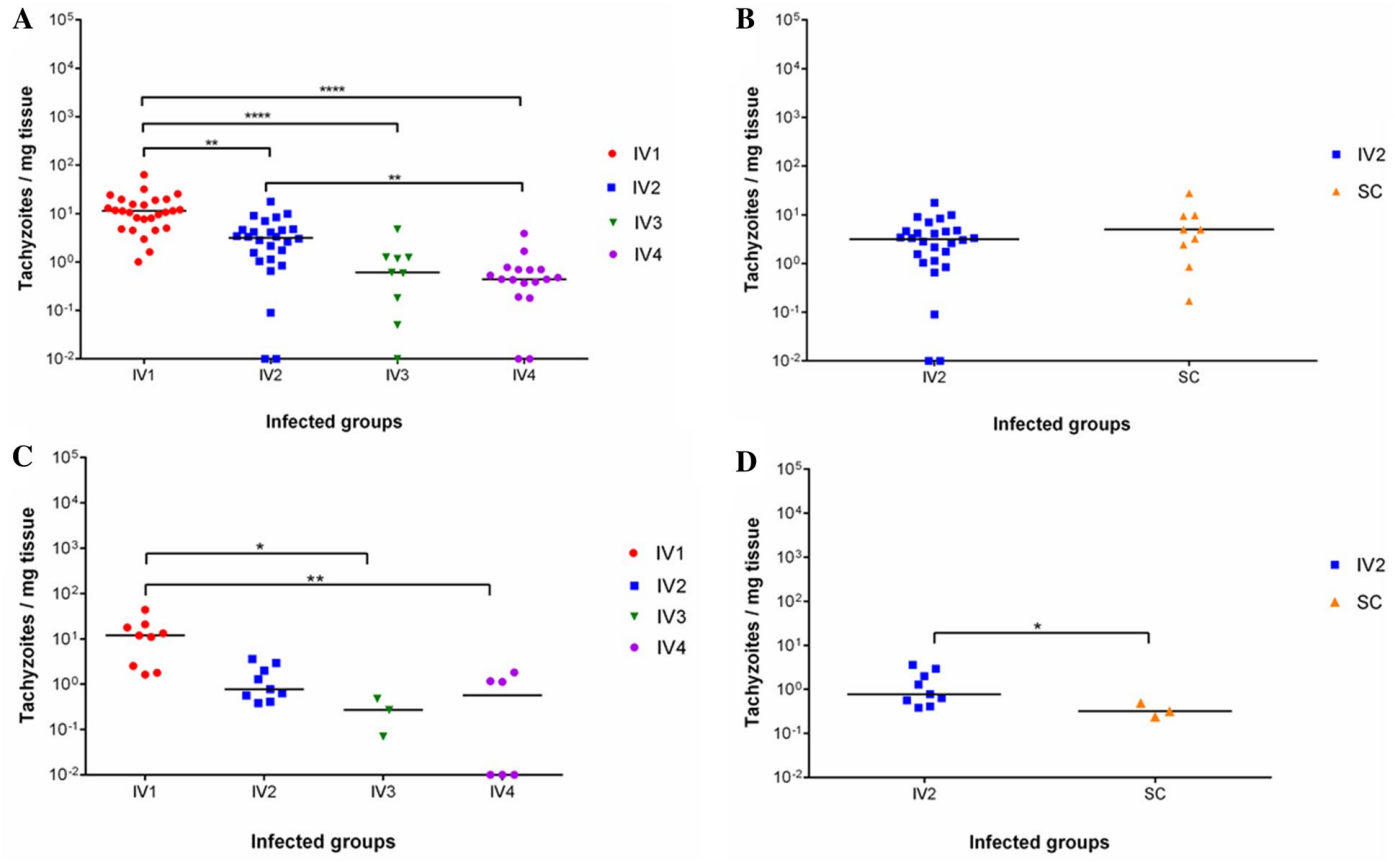
**Dot-plot graphs of the** ***N. caninum*** **burden and foetuses after inoculation with the Nc-Spain7 isolate.** Each dot represents individual values of parasite burden (number of tachyzoites/mg of host tissue), and medians are represented as horizontal lines. Negative burdens (0 parasites) were represented on the log scale as a value below the detection limit of real-time PCR (0.1 tachyzoites) (i.e., 10^−2^). Notice the detrimental parasite burden as the dose decreased (**A**) and the absence of differences for both inoculation routes (IV2 vs. SC, *P* > 0.05) (**B**) for placental tissues, while the parasite burden in foetal brain samples was negatively affected by dose reduction (**C**) as well as by subcutaneous inoculation (SC) (**D**). ****P < 0.0001, **P < 0.01 and *P < 0.05.

*Neospora caninum* DNA was widely detected in all placental tissues of infected heifers that could be investigated (10/10) (Table 1). The frequency of detection of parasite DNA in placentome samples was 100.0% in IV1 (27/27), 92.3% in IV2 (24/26), 88.9% (8/9) in IV3 and 88.9% (16/18) in IV4, with no significant differences in parasite detection in placentomes for the challenge dose. Detection was 100.0% (9/9) in SC, showing an absence of differences due to the route of inoculation (*P* > 0.05). Parasite loads in placentomes significantly decreased with the dose (IV1 > IV2 > IV3 > IV4: *P* < 0.01 (Figure 3A). No variation in parasite loads in placental tissues was associated with the route of inoculation (IV2 vs. SC) (*P* > 0.05) (Figure 3B).

*Neospora caninum* DNA was detected in eight of the ten foetal abortion brains that were investigated (Table 1). In particular, parasite DNA was detected in 100.0% of foetal brain samples from IV1 (9/9) and IV2 (9/9) and in 50.0% of those from the IV4 (3/6) group (Table 1). No positive results were found for the brain samples of the foetus from the IV3 group. Significant differences in parasite DNA detection rates in foetal brain samples were found for the challenge dose (IV1 = IV2 > IV3: *P* < 0.01; IV1 = IV2 > IV4: *P* < 0.05). Foetus from IV3 group with a negative PCR result showed characteristic neosporosis lesions (see below) and was reanalysed by qPCR. As shown in Figure 3C, the parasite burden in the foetal brain was lower in IV3 (*P* < 0.05) together with the loads in IV4 (*P *< 0.01) than those in IV1. Although no significant differences in parasite DNA detection rates in foetal brain samples were found for the route of inoculation (IV2: 9/9, SC: 3/3. *P* > 0.05), the comparison of parasite loads in foetal brains between IV2 and SC groups was higher for the former (*P *< 0.05) (Figure 3D). Scarce parasite DNA detection was found in foetal liver samples from the IV2 (1/9) and SC (1/3) groups, with no effect on route (*P* > 0.05) (Table 1). Additionally, both positive liver samples had a low level of parasite burden (< 0.5 tachyzoites/mg tissue).

Of the 18 calves from infected heifers, *N. caninum* DNA was only detected in brain samples from three animals, one from the IV1 group and two from the IV4 group (Table 1). The parasite load (mean ± SD) for the calf from the IV1 group was 0.5 ± 0.2 tachyzoites/mg tissue; however, no quantification of parasite load in the brain could be performed for the two calves from the IV4 group because the loads were below the detection limit of the technique (0.1 tachyzoites). In calves, neither the challenge doses nor the routes of inoculation were found to be associated with *N. caninum* DNA detection in brain tissues (*P* > 0.05). None of the 18 calves born from infected heifers had *N. caninum* DNA-positive liver samples (Table 1). As expected, all calves from the control group had negative results for brain and liver samples in the PCR assay.

### Lesions in maternal, foetal and calf tissues

**Figure 4:**
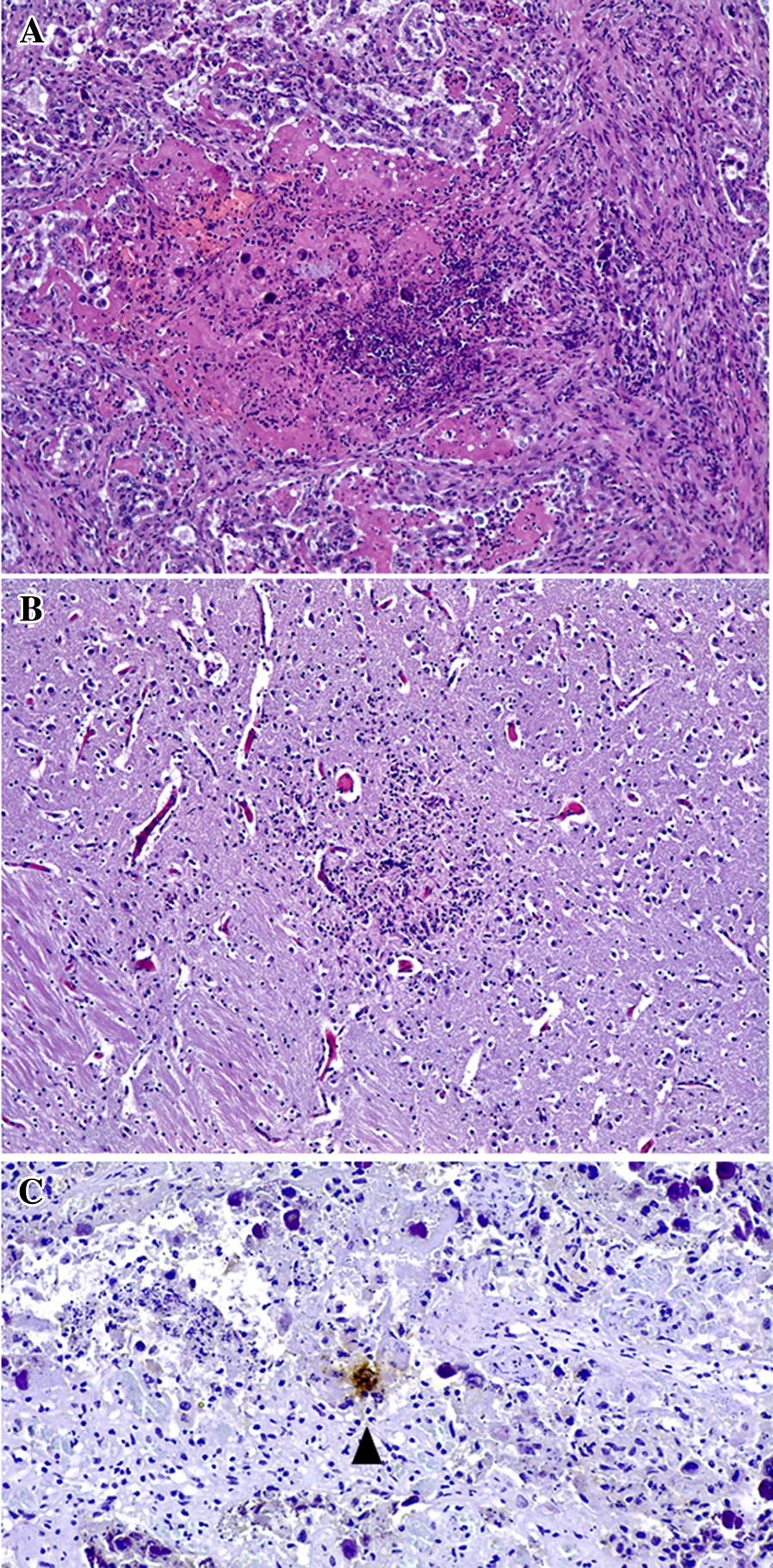
***Neospora caninum*** **specific histological findings. A** Placenta. Focus of necrosis in the interdigitate area of the placentome with abundant serum leakage and infiltration of inflammatory cells in the periphery. Multifocal mineralization if found within the necrosis. ×20 objective. HE. **B** Foetal brain. Glial foci with axonal swelling and degeneration at the center. ×20 objective. HE. **C** Parasitophorous vacuole-like structure labelled at the placentome (filled triangle). ×20 objective. IHC.

All aborted foetuses from the IV-infected groups (IV1 to IV4) showed characteristic lesions of *N. caninum* infection (Table 1). In the placenta, there was a multifocal necrotic placentitis characterized by multiple areas of necrosis at the interdigitated area of the placentome and mild infiltration of non-purulent inflammatory cells in the areas adjacent to the foci (Figure 4A). All the cases where characteristic lesions were found at the placenta also showed non-purulent encephalitis denoted by the presence of necrotic glial foci randomly distributed in the brain (Figure 4B). *N. caninum* antigen was confirmed, either as particulate antigen or as intracellular parasitophorous vacuoles, in relation to these lesions. Lesions in the brain and placenta were subjectively classified as more severe, in terms of size and extension of necrosis, in the IV1 group than in the rest of the groups, with no evident differences among the latter. The other foetal organs evaluated (i.e., liver, skeletal muscle, heart and lung) showed no lesions or antigen labelling. Similarly, there were no lesions or immunohistochemical labelling of the parasite in the only foetus aborted from the subcutaneously infected group (SC). No histological changes, or parasite labelling, were found in any of the calves born from the IV- or SC-infected dams (Table 1) or from the control group.

### Cell-mediated immune response in heifers: IFN-γ production

**Figure 5:**
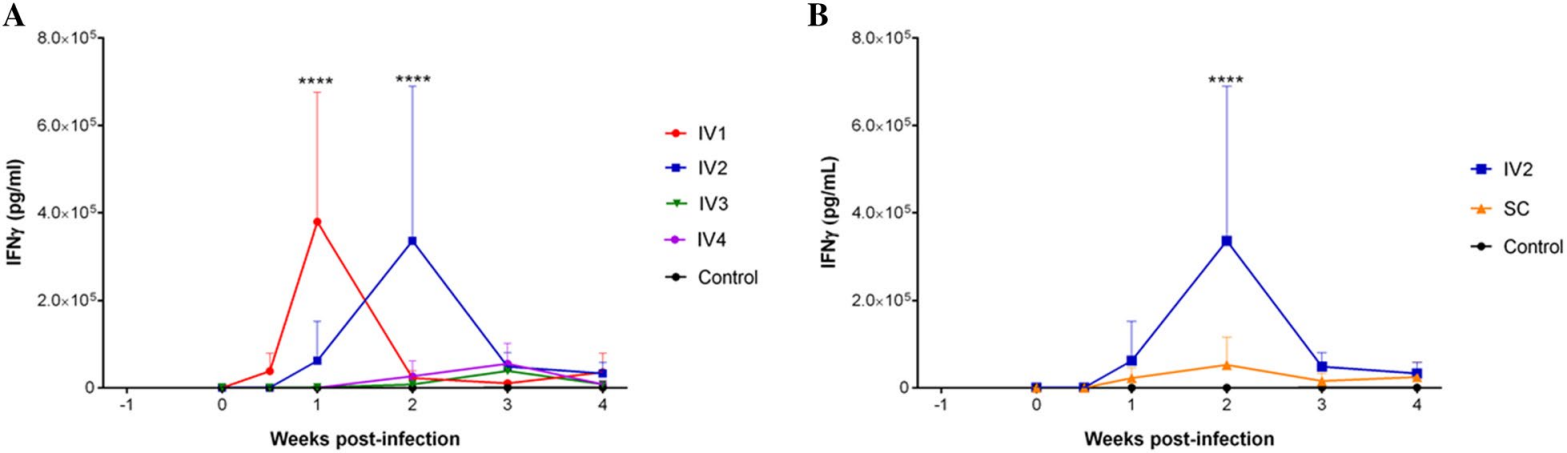
**IFN-γ production after inoculation with the Nc-Spain7 isolate.** Concentrations of IFN-γ, in response to *N. caninum* soluble extract antigen, in lymphocyte culture supernatants of heifers intravenously challenged with 10^7^ (IV1), 10^5^ (IV2), 10^3^ (IV3), and 10^2^ (IV4) tachyzoites and the uninfected control group (**A**), and intravenously (IV2) and subcutaneously (SC) challenged heifers with 10^5^ Nc-Spain7 tachyzoites and the uninfected control group (**B**). Each point represents the mean log IFN-γ concentration (pg/mL) + SD for each group from 0 to 4 wpi. Notice an enhanced IFN-γ production for IV1 (1 wpi (7 dpi)) and IV2 (2 wpi) groups compared to their basal pre-infection levels (**A**) and for the intravenous route (**B**). ****P < 0.0001.

As shown in Figure 5A, the two groups intravenously inoculated with the highest doses of tachyzoites had a significant increase in IFN-γ production on 7 dpi (IV1) and 2 wpi (IV2) compared to those on 0 dpi (*P* < 0.0001). Significant variations in the levels of this proinflammatory cytokine were also associated with the challenge dose. The IV1 group had more elevated IFN-γ concentrations at 7 dpi than the other IV groups (IV1 > IV2 > IV3 > Control > IV4, *P* < 0.0001). At 2 wpi, IFN-γ levels in the IV2 group were higher than those in the IV1, IV3, and IV4 groups and the control group (IV2 > IV4 > IV1 > IV3 > Control, *P* < 0.0001). In contrast, no changes in IFN-γ levels were detected for the IV3 and IV4 groups in comparison to these levels in the control group (*P* > 0.05).

Regarding the route, IV2 led to a significant increase in the IFN-γ levels in the second wpi in comparison with SC challenge for the same dose (*P* < 0.0001) (Figure 5B). Compared with the control group, the levels of IFN-γ were not significantly higher for the SC group at the same time (2 wpi) (*P* > 0.05) (Figure 5B).

### Humoral immune response in heifers, foetuses and calves: IgG antibodies

**Figure 6:**
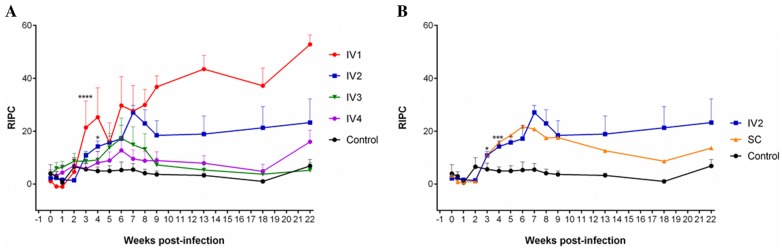
***Neospora caninum*-specific IgG responses after inoculation with the Nc-Spain7 isolate.** IgG response in the serum of heifers intravenously challenged with 10^7^ (IV1), 10^5^ (IV2), 10^3^ (IV3), and 10^2^ (IV4) tachyzoites and the uninfected control group (**A**), and intravenously and subcutaneously challenged heifers with 10^5^ Nc-Spain7 tachyzoites and the uninfected control group (**B**). Total IgG antibodies are expressed as a relative index percent (RIPC). RIPC value for each sample was estimated according to the following formula from corresponding optical densitity (OD) values: RIPC = [(OD sample − OD_405_ negative control)/(OD_405_ positive control − OD_405_ negative control)] × 100. Each point represents the mean + SEM (Standard error of mean) at the different sampling times until the endpoint for each group. Note that the IV1 group had a peak of IgG levels by 3 wpi compared to its basal pre-infection levels (*P* < 0.0001), and the IV2 group had a significant increase in IgG levels 1 week later (4 wpi) (*P* < 0.05) (**A**). Note the delayed increase in IgG levels with respect to basal pre-infection levels for the SC group compared to the IV2 group (*P* < 0.001) (**B**). ****P < 0.0001, ***P < 0.001 and *P < 0.05.

*Neospora caninum*-specific serum IgG antibody responses are presented in Figure 6. With regard to the challenge dose (Figure 6A), the IV1 and IV2 groups showed increased IgG levels at 3 and 4 wpi, respectively, compared to their basal pre-infection levels (IV1: *P* < 0.0001; IV2: *P* < 0.05). IgG levels increased significantly in IV1 at 3 and 4 wpi with respect to the control group (3 wpi: *P* < 0.05; 4 wpi: *P* < 0.01). A reduction in IgG levels was associated with decreased infectious doses at 3 and 4 wpi (3 wpi: IV1 > IV4 > Control: *P* < 0.05; 4 wpi: IV1 > IV3 > IV4 > Control: *P* < 0.05). From the 4th week until the end point of the experiment, a dose effect on mean IgG levels was also detected in the IV1 and IV2 groups (IV1 > IV2 > IV3 > IV4 > Control, *P* < 0.001; IV2 > IV3 > IV4 > Control, *P* < 0.5).

No route effect was found to be statistically significant during the studied period (*P* > 0.05) (Figure 6B). In the early stages of infection (up to 4 wpi), both routes had a significant increase in IgG levels compared to their corresponding basal pre-infection levels. In particular, at 3 wpi for the IV2 group (*P* < 0.05) and 1 week later for the SC group (*P* < 0.001). Both IV2 and SC had significantly higher IgG levels than the control group at 4 wpi (*P* < 0.05) and afterwards (IV2 > Control, *P* < 0.0001; SC > Control, *P* < 0.0001). Non-infected heifers (Control) had mean RIPC values below the cut-off value (Figure 6).

*Neospora*-specific IgG antibody responses were also analysed in foetal fluids and precolostral sera from newborn calves (Table 1). Seropositive titres, ranging from 16 to 32, were only detected in two of the eight fluids of foetuses that could be collected, both corresponding to IV groups and foetal death occurring at 39 (IV2) and 62 (IV4) dpi. Seropositivity against *N. caninum* of calves was found in the IV1 (2/2), IV2 (2/3) and IV3 (1/5) groups, with IFAT titres ranging from 6400 (IV2) to 50 (IV3) (Table 1). Although the reduced challenge dose was not associated with a decrease in categorical IFAT results (*P* > 0.05), it was correlated with IFAT titres (IV1 vs. IV3: *P *< 0.05) (Table 1). There were no significant differences in the antibody titre of precolostral sera for IV and SC routes (IV2 vs. SC: *P *> 0.05).

## Discussion

The standardization of models of bovine neosporosis, considering variables such as the stage of gestation, the parasite isolate, the challenge dose and the route of inoculation, remains a challenging and high-priority issue to obtain a better understanding of the immunopathological features of host-parasite interactions and to have reliable tools to evaluate drugs and vaccine candidates [19, 20]. Pregnant ruminant models during mid-pregnancy, with sheep (90 dg) and cattle (110 dg) primo-infected with the virulent Nc-Spain7 isolate, have been proven to be relevant models to evaluate exogenous transplacental transmission of *N. caninum* [19]. However, limited research has been performed in cattle for this well-characterized isolate. In fact, there are only three bovine studies using IV inoculation with doses of 10^8^ [15] and 10^7^ tachyzoites [16, 18], and the SC route has not been tried so far. Herein, we present a study that examines the clinical outcome, parasite transmission and immune effects of four challenge doses and two routes of inoculations (IV and SC) of the virulent Nc-Spain7 isolate at mid-gestation (110 dg).

It is well known that the clinical outcome can vary with the tachyzoite infective dose; therefore, dose-titration studies are also necessary to contribute to the refinement and standardization of a bovine model [21, 22]. Foetal damage is a crucial parameter to be considered in any experimental models of bovine neosporosis [20]. Recently, foetal death, between 2 and 6 wpi, was demonstrated in three out of six heifers intravenously inoculated with a dose of 10^7^ tachyzoites of the Nc-Spain7 isolate at mid-gestation [18]. Consistent with this finding, our study showed that lower doses, up to 10^2^ tachyzoites, can also induce foetal death at this stage of gestation. These data also underline differences between host species, with the infection with the Nc-Spain7 isolate being more aggressive for sheep, since IV inocula of 10^6^ and 10^5^ tachyzoites led to 100% ovine abortion rates [22, 25]. In turn, bovine foetal death ranged from 66.7 to 50.0% for doses of 10^7^ and 10^5^ tachyzoites, respectively. Moreover, the challenge dose responsible for abortion in 50% of infected ewes was as low as 10^2^ tachyzoites [22], while doses at least threefold higher (10^5^) are needed to reach these rates in cattle. Foetal death presentation mostly occurred between 4 and 6 wpi, as already proposed [18], although one foetal death, associated with the lowest dose, was delayed until 9 wpi. In common for bovine and ovine species, prolonged median survival times could be associated with decreasing doses [22, 32]. However, the impact of sample size in the present study could be responsible for the absence of statistical significance for foetal death when the four doses were evaluated.

Rectal temperature is another clinical parameter of interest for monitoring *N. caninum* infections in ruminants. In this regard, a transient or a biphasic rise of body temperature during the first wpi as previously proposed [20] was herein confirmed only in heifers intravenously challenged with the highest dose (IV1). Our results confirm an earlier febrile response associated with infections in the second term of gestation (3–5 dpi) [18] compared to those observed in the first term of gestation (5–7 dpi) [16]. However, in our study, the fever was less persistent than in the two studies mentioned above, probably in relationship with other host-related factors (i.e., breed) than parasite-dependent variables. As the dose decreased, the mean rectal temperatures remained below 39.5 °C. Thus, it could be argued that the intensity of the temperature response varies depending on the number of tachyzoites for a given isolate that are replicating in the host, as already proposed [20]. The initial multiplication of tachyzoites after the inoculation of low doses could be controlled by the host immunity; however, the capacity of the highest dose (10^7^) to surpass the host immune defences could allow a second round of parasite multiplication as a result of the biphasic rise in the temperature after IV inoculation observed in the IV1 group. It has also been demonstrated that reduced doses of tachyzoites of the Nc-Spain7 isolate can cause a delay in the fever peak in pregnant sheep at mid-gestation [22].

The detection and quantification of *N. caninum* DNA and the observation of tissue damage are key indicators of parasite multiplication, transmission and dissemination [20]. In the present study, in addition to placental tissue, foetal brain and liver samples were investigated for parasite transmission and histopathological techniques. Parasite DNA was detected in all placental tissues irrespective of the dose (IV1, IV2, IV3, and IV4). Interestingly, our results showed marked differences in parasitological distribution between foetal death cases and calves born from infected heifers (Table 1). *N. caninum* DNA-positive foetal brain samples were detected for all experimental IV groups by nested ITS1-PCR, except for the foetus from IV3 group (Table 1). These findings could be explained by the low parasite loads quantified by qPCR after its reanalysis. The parasite load significantly decreased for doses of ≤ 10^5^ tachyzoites (Figure 3A), which underlines the effect of the parasite dose on infection outcome, as discussed above for the pattern of biphasic rise in temperature. On the other hand, the low parasite DNA detection (Table 1) and load (< 0.5 tachyzoites/mg tissue) in foetal liver samples (Table 1) corroborated the tropism of *N. caninum* described for foetal brain tissue [18]. *N. caninum* DNA-positive brain calf samples were limited to three animals (IV1: *n* = 1, and IV4: *n* = 2) (Table 1), with a lower parasite burden as the dose decreased (IV1 > IV4). This is a common pattern observed in other models aimed at studying vertical transmission at middle and late gestational stages that has been associated with the ability of the foetus at this stage to limit parasite multiplication by means of an immunocompetent responses [20]. Unlike histopathological changes compatible with *Neospora* in foetuses, no lesions were found in any calves. It should be noted, however, that even the lowest dose of parasite (10^2^ tachyzoites) led to an efficient vertical transmission of the parasite, since it was possible to detect *N. caninum* DNA in two of the three calves (Table 1). In addition, we detected parasite DNA in the foetal liver longer post infection than previously described in similar experimental conditions. Furthermore, these results show large differences with high parasite burdens and lesions in liver samples of foetuses aborted from heifers challenged at 70 dg [16], highlighting the importance of the time point at the primo-infection in terms of transmission and the development of foetal lesions in tissues. Therefore, the absence of a foetal immunocompetence during early gestation could enable pathogen invasion and dissemination, while foetal immunocompetence increases at mid-gestation and results in variable clinical outcomes [19, 20].

In a context where the placenta plays a key role in allowing or restraining the spread of *N. caninum* infection [17, 33] and where abortions can occur if severe lesions develop in placental tissues [17], it is remarkable that the dose of 10^2^ tachyzoites was enough to result in lesions compatible with *N. caninum* infection. Furthermore, the cotyledonary synepitheliochorial placenta in ruminants [34] does not allow the passage of maternal IgG antibodies to the foetus [18, 35], and therefore, the presence of antibodies in foetal fluid and in precolostral serum samples are indicative of the successful vertical transmission of *N. caninum* in abortions and newborn calves, respectively [36]. Interestingly, we identified the presence of *N. caninum* antibodies in precolostral sera of calves birthed from infected heifers with doses as low as 10^3^ tachyzoites (IV3). One plausible explanation for vertical transmission is that it could be favoured by decreased IFN-γ and antibody-mediated immune responses. It has also been suggested that there is a correlation between time at foetal death and *Neospora* IFAT titres, regardless of the parasite isolate and the time of gestation when infected. The presence of two foetuses with IgG-IFAT titre ≥ 16 positive by 6–9 wpi was consistent with data reported by others after experimental infection with Nc-Spain7 isolate at early and mid-gestation. According to these studies, *N. caninum* antibodies were found in foetal bovine serum or fluids during the same period of time or even starting earlier (at 2 wpi) when infected with a higher inoculum (10^8^ tachyzoites) [15–18]. However, the titres were associated with IV doses lower than those already published. In addition, it should be noted that foetal immunocompetence begins to develop at approximately 100 dg, that foetal lymphocytes are able to respond to T cell mitogens and allo-antigen stimulation by 120 dg [37, 38], and that the development of the innate immune responses mediated by phagocytic cells is not fully developed until late gestation with low levels of IFN-γ and other components [19]. Unlike aborted foetuses, high frequencies of seropositivity were found for group IV1 (100%) and IV2 (66.7%) calves. In fact, there was an association between the challenge dose and IFAT titres in precolostral sera. Thus, this enhanced immune response in the calves may be explained by a higher antigen exposure.

Although much still remains to be understood regarding immunopathological events that are involved in *N. caninum*-associated abortions, increased IFN-γ-mediated host immune responses appear to confer partial protection by limiting parasite proliferation, while humoral immunity is more indicative of parasite exposure rather than protection [39, 40]. IV1 and IV2 heifers had increased cell-mediated peripheral immune responses compared to those of the control group at 1 and 2 wpi, respectively; these results are in agreement with those results found after IV inoculation of 10^7^ tachyzoites during early pregnancy (70 dg) in Holstein–Friesian heifers [16]. The fact that the groups that had higher abortion rates (≥ 50%) were those with enhanced IFN-γ productions may indicate that this response was not enough for avoiding infection progression, or on the other hand, that a high number of tachyzoites triggered an exaggerated IFN-γ-mediated response able to cause adverse effects on pregnancy [39–42]. Indeed, cases of IFN-γ-induced abortions have already been demonstrated for the absence of pathogens or antigens in murine pregnancies [43]. For the adaptive immune response to *N. caninum*, IV1 and IV2 heifers had a pattern of seroconversion similar to those already reported for the dose of 10^7^ tachyzoites of the Nc-Spain7 isolate at early [16] and mid-gestation [39], with detectable IgG antibodies by 3–4 wpi (Figure 6A). In contrast, IV3 and IV4 were not related to enhanced IgG antibodies. Thus, 10^7^ and 10^5^ tachyzoites appeared as the threshold doses for *N. caninum*-specific serum IgG antibody responses in cattle.

The route of infection is a key factor that may explain variations in the clinical outcome of infections caused by apicomplexan protozoa, such as *N. caninum* and related *Toxoplasma gondii* [44, 45]. The SC route has been proposed to be more adequate than IV administration for experimental purposes aimed at modelling natural *N. caninum* infections (reviewed by [46, 47]), and there is great consensus on the election of the sub-iliac lymph node as an inoculation site. Several studies have considered the use of the SC route for *N. caninum* isolates (mostly NC1) in experimental pregnant bovine neosporosis models [8, 11, 33, 48], and more recently, the effect of IV and SC inoculation of the Nc-Spain7 isolate (10^4^ tachyzoites) has been investigated in pregnant sheep at mid-gestation (90 dg) [22].

Higher foetal mortality estimations have also been suggested following IV inoculation than SC in cattle in early pregnancy (IV_28 dpi_: 2/2 vs. SC_28 dpi_: 1/2) [11] and sheep at mid-gestation (90 dg) (IV: 4/5 vs. SC: 3/4) [22]. However, the foetal death rate using a dose of 10_5_ Nc-Spain7 tachyzoites was three times higher for the IV (50.0%; 3/6) route than for the SC route (16.7%; 1/6), although no statistically significant route-dependent efect was found in this study, probably due to the sample size of the groups..

Early immune interactions between *N. caninum* tachyzoites and the sub-iliac lymph node, which drains the area of inoculation, are assumed to have occurred during parasite replication in the SC group due to the evident enlargement of this lymph node during the first 3 weeks after SC challenge, as described by others [22, 33]. Although previous descriptions of transient fever associated with the SC inoculation of the NC1 isolate in cattle at mid-gestation (10^7^ or 5 × 10^8^ tachyzoites) [33, 36] have been reported, afebrile responses have also been described for the SC route in early pregnancy [11], as well as for subcutaneously challenged ewes at 90 dg (10^4^ tachyzoites) [22].

With regard to the effect of the route of inoculation on parasite transmission, a similar pattern was observed to that already proposed for sheep placental tissues after the inoculation of a tenfold lower dose by both routes [22]. Interestingly, despite a comparable number of tachyzoites per mg of placentomal tissue attributable to the route of inoculation (IV2 vs. SC), a lower parasite load was found in the foetal brain after SC compared to IV inoculation. It is therefore difficult to draw consistent conclusions about the immunologic response of subcutaneously challenged foetuses to *N. caninum* infection from one case. In this sense, differential factors related to the presentation of antigens [49] may be involved following any of these two routes of inoculation for a similar number of tachyzoites able to reach the placenta. IV inoculation could encourage a more rapid systemic immune response and parasite distribution to target tissues than SC route [14, 49]. This fact might explain why IV inoculation resulted in intensified IFN-γ-mediated peripheral immune responses at 2 wpi (Figure 5B), in accordance with the descriptions of systemic IFN-γ responses (10 dpi) after *N. caninum* IV and SC challenge in ewes at 90 dg [22]. Nonetheless, further research is needed to understand how foetal immunity enables parasite control or, on the contrary, how the maternal–foetal interface is affected by *N. caninum* infections and tachyzoites are able to spread to the foetal brain as well as to other foetal organs and replicate in these tissues.

Whether IgG profiles against *N. caninum* in dams were route dependent was also investigated in our study. A delay of 1 week in the time course of the production of a specific IgG antibody response to *N. caninum* was detected for the SC route (4 wpi) when compared to the IV route (3 wpi), but there were no differences in IgG serum levels between routes of administration when compared at any time, as already published [22].

Hence, a different exposure to *N. caninum* antigens for the SC route may be proposed. No differences in the antibody titre of precolostral sera from newborn calves could be attributed to the type of inoculation (IV2 vs. SC). Nevertheless, high titres of antibodies to *Neospora* in the precolostral sera of calves born to dams subcutaneously infected with 5 × 10^8^ tachyzoites of the NC1 isolate at 140 dg have already been reported, as well as for specific cell-mediated immune responses in PBMCs in these precolostral sera, indicating intra-uterine exposure to the parasite.

In conclusion, inoculation of 10^7^ (IV1) and 10^5^ tachyzoites (IV2) of the virulent Nc-Spain7 isolate led to the highest abortion rates and resulted in specific IgG responses in precolostral sera from newborn calves. Vertical transmission was also proven with considerably lower doses than those already reported in cattle, such as 10^3^ (IV3) and 10^2^ tachyzoites (IV4). Additionally, a dose-dependent effect for parasite load in placental and foetal brain tissues was detected. Regarding the routes of administration, there was a different impact on the clinical outcome, parasite load in foetal brain tissues and lesion development, since evidence of less aggressive infections occurred after SC challenge than IV. In dams, the IFN-γ productions and dynamics of anti-*N. caninum* IgG antibodies varied with the dose, and the cell-mediated immune response was also found to be route-dependent. Therefore, IV inoculation of 10^7^ tachyzoites of the Nc-Spain7 isolate appears as the best dose/route for bovine pathogenesis models because of its high abortion rate and parasite vertical transmission in both foetus and calf. However, further studies are needed to contribute to the refinement and standardization of a bovine neosporosis pregnant model during mid-gestation based on subcutaneous inoculation to evaluate vaccine candidates or drugs for the control of bovine neosporosis.

## Supplementary information

**Additional file 1. Materials and methods.** Detailed description of the (1) health and reproductive management of heifers and (2) molecular (tissue DNA extraction and PCR and qPCR determinations), cellular (peripheral blood stimulation assays and quantification of IFN-γ) and serological analyses (ELISA and IFAT).

**Additional file 2. Rectal temperature records.**

## Data Availability

The datasets supporting the conclusions of this article are included within the article and its additional files.

## Abbreviations

IV: intravenous
SC: subcutaneous
BVD: bovine viral diarrhea
IBR: infectious bovine rhinotracheitis
dg: days of gestation
dpi: days post-infection
wpi: weeks post-infection
PBS: phosphate-buffered saline
qPCR: real-time polymerase chain reaction
HE: haematoxylin–eosin
ELISA: enzyme-linked immunosorbent assay
