# Correction to: Effects of challenge dose and inoculation route of the virulent *Neospora caninum* Nc-Spain7 isolate in pregnant cattle at mid-gestation

**DOI:** 10.1186/s13567-019-0700-9

**Published:** 2019-10-14

**Authors:** Patricia Vázquez, Koldo Osoro, Miguel Fernández, Alicia Román-Trufero, Javier Regidor-Cerrillo, Laura Jiménez-Pelayo, Marta García-Sánchez, Silvia Rojo-Montejo, Julio Benavides, Pilar Horcajo, Luis Miguel Ortega-Mora

**Affiliations:** 1 SALUVET, Animal Health Department, Faculty of Veterinary Sciences, Complutense University of Madrid, Ciudad Universitaria s/n, 28040 Madrid, Spain; 2 Regional Service for Research and Agri-Food Development (SERIDA), 33300 Villaviciosa, Asturias Spain; 3 Mountain Livestock Institute, Animal Health Department, University of León CSIC-ULE, 24346 Grulleros, León Spain; 4 SALUVET-Innova S.L., Faculty of Veterinary Sciences, Complutense University of Madrid, Ciudad Universitaria s/n, 28040 Madrid, Spain

## Correction to: Vet Res (2019) 50:68 10.1186/s13567-019-0686-3

In the original publication of this article [1], there are errors in the Figure 5, the “ml” should be replaced by “mL” and “IFNγ” should be “IFN-γ” in panels A and B. The corrected Figure 5 is given in this correction.

**Figure 5:**
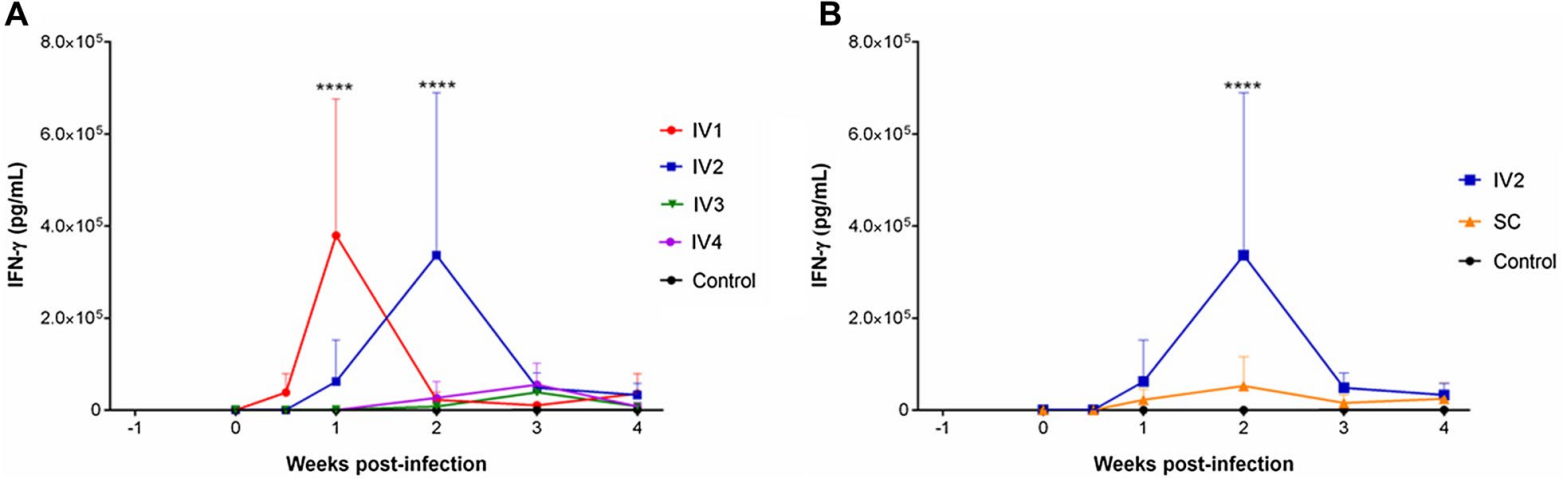
**IFN-γ production after inoculation with the Nc-Spain7 isolate.** Concentrations of IFN-γ, in response to *N. caninum* soluble extract antigen, in lymphocyte culture supernatants of heifers intravenously challenged with 10^7^ (IV1), 10^5^ (IV2), 10^3^ (IV3), and 10^2^ (IV4) tachyzoites and the uninfected control group (**A**), and intravenously (IV2) and subcutaneously (SC) challenged heifers with 10^5^ Nc-Spain7 tachyzoites and the uninfected control group (**B**). Each point represents the mean log IFN-γ concentration (pg/mL) + SD for each group from 0 to 4 wpi. Notice an enhanced IFN-γ production for IV1 (1 wpi (7 dpi)) and IV2 (2 wpi) groups compared to their basal pre-infection levels (**A**) and for the intravenous route (**B**). ****P < 0.0001.

In the main text (on page 11), the sentence ‘However, the foetal death rate using a dose of 10_5_ Nc-Spain7 tachyzoites was three times higher for the IV (50.0%; 3/6) route than for the SC route (16.7%; 1/6),…’ should be ‘10^5^ Nc-Spain7 tachyzoites’ here instead of ‘10_5_ Nc-Spain7’.

The original publication has been corrected.
