# Supplementary material for: Effects of challenge dose and inoculation route of the virulent Neospora caninum Nc-Spain7 isolate in pregnant cattle at mid-gestation
Source: Vet Res. 2019 Sep 23;50:68. doi: 10.1186/s13567-019-0686-3 (PMC6755697; doi:10.1186/s13567-019-0686-3)
Supplement: Supplementary file 1 — Additional file 1. Materials and methods. Detailed description of the (1) health and reproductive management of heifers and (2) molecular (tissue DNA extraction and PCR and qPCR determinations), cellular (peripheral blood stimulation assays and quantification of IFN-γ) and serological analyses (ELISA and IFAT). [file 13567_2019_686_MOESM1_ESM.docx]

**Additional file 1 Materials and methods**

## Health and reproductive management of heifers

The administration of antiparasitic products (Endoex® and Albendex®, s.p. veterinaria, s.a., Riudoms, Spain; Animec Plus®, Cenavisa laboratorios, Tarragona, Spain) and the implementation of vaccine protocols against bovine respiratory disease (BOVILIS BOVIPAST® RSP, MSD Animal Health, Milton Keynes, UK) and IBR and BVD viruses (Bovilis® IBR Marker Live, MSD Animal Health, Milton Keynes, UK; Bovilis® BVD, MSD Animal Health, Milton Keynes, UK) were performed following the manufacturer's recommendations.

Heifers were periodically weighed to confirm that they had appropriate body weights to start the oestrus synchronization protocol. Briefly, the protocol consisted of the insertion of a commercial intravaginal progesterone releasing sponge (CIDR, Zoetis, New Jersey, USA) and IM administration of 100 µg of synthetic gonadorelin (GnRH) (Cystoreline^®,^ CEVA Santé Animale, Libourne, France) on day 0, CIDR removal and IM administration of 400 UI of equine serum gonadotropin (Folligon®, MSD Animal Health, Milton Keynes, UK) and 25 mg of synthetic prostaglandin F2α (PGF 2α) analogue (Dinolytic®, Zoetis, New Jersey, USA) on day 5, and a second IM administration of 25 mg of PGF 2α analogue on day 6. Fifty-six hours after administering the first dose of PGF 2α analogue, heifers were artificially inseminated and received a second dose of GnRH (100 µg) (Cystoreline^®^, CEVA Santé Animale, Libourne, France) (day 7). Semen from two Asturiana de la Montaña registered bulls (Spanish Association of breeders of selected cattle of the Asturiana de la Montaña breed, ASEAMO) were used for calving ease. Visual heat detection was performed twenty-one days after artificial insemination. The detection of early pregnancy-associated glycoproteins (PAGs) in serum samples with the commercial IDEXX Bovine Pregnancy Test (IDEXX Laboratories Inc., Westbrook, USA) and transrectal ultrasonography (US) were used to confirm pregnancy and foetal viability on day 35 after artificial insemination.

## Tissue DNA extraction and *N. caninum* DNA detection and quantification by PCR determinations

*DNA extraction and determination of DNA concentration*

Genomic DNA extraction from tissue samples using the commercial Maxwell® 16 Mouse Tail DNA Purification Kit, developed for automated Maxwell® 16 System (Promega, Wisconsin, USA), was performed following the manufacturer’s recommendations. In each round at least one sample from the control group was included to avoid cross-contamination during this step. Concentration of DNA was determined for each sample using Synergy™ H1 multi-mode microplate reader (Biotek, Winooski, USA) and Gen5 version 2.09.1 software (Biotek, Winooski, USA), and adjusted to 100 ng/µL in RNase, DNase, and protease free water.

*Parasite DNA detection by nested-ITS1 PCR*

Neospora DNA detection was carried out by a nested-PCR adapted to a single tube for the amplification of the internal transcribed spacer (ITS1) region of *N. caninum* using external primers (TgNN1-TgNN2) and internal primers (NP1–NP2), as previously described [16, 27, 28]. Nested ITS1-PCR was carried out, from 5 μL of sample DNA, in nine samples of the placentomes in aborted heifers, and three and ten samples for foetal and calf tissues, respectively. Each ITS1-PCR assay included samples from the uninfected group, two negative controls (NC1: 20 μL mix and NC2: 20 μL mix + 5 μL molecular grade water) and six positive controls with *N. caninum* genomic DNA equivalent to 10 (*n* = 1), 1 (*n* = 3) and 0.1 (*n* = 2) tachyzoites in 100 ng of sheep genomic DNA. 10-15 μL aliquots of the nested ITS1-PCR products were visualized under UV light in 1.5% agarose gel stained with GelRed™ nucleic acid gel stain (Biotium Inc., Fremont, USA) to detect the *N. caninum*-specific 247 bp amplification product. Validation of each nested ITS1-PCR assay required the absence of amplification of samples from the uninfected group and negative controls, and the amplification of at least two positive controls (10 and 1 tachyzoites).

*Parasite DNA quantification by real time PCR (qPCR)*

Positive ITS1 DNA samples detected by nested-PCR from tissues were quantified by using real-time PCR (qPCR) and the [ABI 7500](https://www.google.es/url?sa=t&rct=j&q=&esrc=s&source=web&cd=1&cad=rja&uact=8&ved=0CCAQFjAAahUKEwiJ8pG73_HIAhUBChoKHXxSCEo&url=http%3A%2F%2Fwww.appliedbiosystems.com%2Fabsite%2Fus%2Fen%2Fhome%2Fsupport%2Fsoftware%2Freal-time-pcr%2Fab-7300.html&usg=AFQjCNGcK_ltiuLnd7Uh0CxqpNxVxouk6Q) Fast Real-time PCR System (Applied Biosystems, Foster City, CA, USA). Briefly, a volume of 5 μL of DNA (adjusted to 20 ng/µL) from each sample was used for the qPCR assays, using Go Taq® qPCR Master Mix (Promega, Wisconsin, USA), for parasite quantification from *N. caninum* Nc-5 sequence [29] and host DNA from the β-actin gene [30]. As proposed by [16], the number of *N. caninum* tachyzoites was determined by interpolating the corresponding cycle threshold (Ct) values on the two standard curves: one ranging from 10^-1^ to 10^5^ tachyzoites (10-fold serial dilutions in a solution of bovine genomic DNA), and the other curve of 320 ng, 160 ng, 80 ng, 40 ng, 20 ng, 4 ng and 1 ng of bovine genomic DNA. Both standard DNA curves showed good efficiency: R^2^ > 0.99, average slope of *N. caninum* and β-actin standard curves: −3.4. Parasite burden was expressed as tachyzoite number / mg bovine tissue.

## Peripheral blood stimulation assay and quantification of interferon-gamma (IFN-γ) release

Heparinized blood samples were cultured in 24-well flat-bottom plates (Thermo Fisher Scientific, Waltham, USA) within 2-8 hours of collection. Briefly, 500 µL of blood were added to 500 µL of RPMI 1640 medium (Gibco, Paisley, UK) supplemented with 10% foetal bovine serum (Thermo Fisher Scientific, Waltham, USA) and 1% bacteriostatic and bactericidal Peniciline-Estreptomicine-Anfoterincine B solution (100X) (Lonza, Belgium). Next, samples were cultured in duplicate with *N. caninum* soluble extract antigen at 5 μg/mL [23], concanavalin (ConA) (Sigma-Aldrich, Madrid, Spain), at 5 μg/mL as positive control, and PBS as negative control. After 24 h of incubation (Temperature: 37 °C, CO_2_ level: 5%, humidity: 100%), plates were centrifuged at 1000 × *g* for 10 min at 4 °C. Supernatants were stored at −80 °C until IFN-γ release was measured with a commercial bovine IFN-γ ELISA kit (Mabtech AB, Nacka Strand, Sweden), following the manufacturer’s guidelines.

## Serological analyses: *N. caninum*-specific IgG responses

*Detection of IgG responses in heifers and calves by an in-house indirect ELISA*

Briefly, 96-well microtiter plates (Thermo Fisher Scientific, Waltham, USA) were coated with soluble *N. caninum* antigen [23] (0.5 µg/mL in carbonate buffer (100 mM, pH 9.6)). After overnight coating at 4 °C, plates were washed with phosphate buffer saline (PBS) (pH 7.4) containing 0.05% Tween 20 (PBS-T) thrice. In order to avoid non-specific binding, plates were blocked with 300 μL of blocking solution (PBS-T containing 5% horse serum [SHI]) and incubating for two hours at room temperature. In each plate, 100 µL of sera samples and duplicate controls (negative and positive) diluted 1:100 in block solution were incubated for 1 h at 37 °C. After a washing step with PBS-T (x3), 100 μL of anti-bovine IgG peroxidase conjugate (Sigma-Aldrich, Madrid, Spain) diluted 1:10000 in block solution was added and incubated for 1 h at 37 °C. After a further wash step with PBS-T (x3), ABTS substrate (Roche, Basilea, Switzerland) was added (100 μL per well) and the reaction was stopped with 0.3 M oxalic acid (100 μL per well) when the optical density, read at 405 nm, (OD_405_) of the positive control was close to 1.1. A relative index percent (RIPC) was estimated for each sample according to the following formula: RIPC = [(OD sample – OD_405_ negative control) / (OD_405_ positive control –OD_405_ negative control)] × 100. Bovine serum samples with RIPC values greater than or equal to 12.09 are considered positive. Negative samples are associated with RIPC values less than 6. And doubtful results correspond to those samples with IRPC values greater than 6 and less than 12.09.

*Detection of IgG responses in foetal fluids and precolostral sera by the indirect fluorescent antibody test (IFAT)*

*N. caninum*-specific IgG responses in foetal fluids and precolostral sera were measured by indirect fluorescent antibody test (IFAT), as previously described [23]. Samples were diluted at two-fold serial dilutions in PBS 1x, starting at 1:8 for foetal fluids and 1:50 for precolostral sera, up to the end point titre. Visualization of unbroken tachyzoite membrane fluorescence at titre ≥8 for foetal fluids or ≥ 50 for precolostral sera was considered a positive reaction.
