# Supplementary material for: Effects of challenge dose and inoculation route of the virulent Neospora caninum Nc-Spain7 isolate in pregnant cattle at mid-gestation
Source: Vet Res. 2019 Sep 23;50:68. doi: 10.1186/s13567-019-0686-3 (PMC6755697; doi:10.1186/s13567-019-0686-3)
Supplement: Supplementary file 2 — Additional file 2. Rectal temperature records. [file 13567_2019_686_MOESM2_ESM.docx]

| **ID** | **Time monitoring of rectal temperature** | | | | | | | | | | | | | | | | | | | | | |
| --- | --- | --- | --- | --- | --- | --- | --- | --- | --- | --- | --- | --- | --- | --- | --- | --- | --- | --- | --- | --- | --- | --- |
|  | **Days post-infection** | | | | | | | | | | | | | | | **Weeks post-infection** | | | | | | |
|  | **0** | **1** | **2** | **3** | **4** | **5** | **6** | **7** | **8** | **9** | **10** | **11** | **12** | **13** | **14** | **3** | **4** | **5** | **6** | **7** | **8** | **9** |
| **IV1.1** | 38.3 | 38.7 | 38.6 | 40.3 | 38.6 | 38.9 | 38.4 | 39.0 | 38.5 | 38.5 | 38.0 | 38.3 | 38.4 | 38.4 | 38.4 | 38.3 | 38.8 |  |  |  |  |  |
| **IV1.2** | 38.7 | 39.3 | 39.5 | 40.3 | 39.1 | 39.2 | 38.5 | 38.7 | 38.9 | 38.9 | 38.4 | 39.6 | 39.5 | 38.7 | 38.0 | 38.8 | 38.6 |  |  |  |  |  |
| **IV1.3** | 38.8 | 39.4 | 38.8 | 39.6 | 38.4 | 39.0 | 38.6 | 39.2 | 38.9 | 38.5 | 38.6 | 38.0 | 38.6 | 38.7 | 38.6 | 38.0 | 38.7 |  |  |  |  |  |
| **IV1.4** | 38.6 | 40.1 | 39.1 | 39.3 | 38.9 | 38.1 | 38.4 | 38.5 | 38.3 | 38.2 | 38.2 | 37.8 | 38.4 | 38.3 | 38.6 | 37.9 | 38.6 | 39.1 | 38.6 | 39.0 | 38.6 | 38.3 |
| **IV1.5** | 38.5 | 39.7 | 39.3 | 39.7 | 39.8 | 38.9 | 39.2 | 38.9 | 39.0 | 38.7 | 37.9 | 39.3 | 38.6 | 37.6 | 38.2 | 38.5 | 38.7 | 38.9 | 38.8 | 39.0 | 38.5 | 38.8 |
| **IV1.6** | 38.8 | 39.7 | 40.8 | 40.3 | 38.3 | 37.9 | 38.3 | 38.8 | 38.5 | 38.7 | 38.1 | 38.3 | 38.4 | 38.3 | 38.2 | 38.5 | 38.4 |  |  |  |  |  |
| **IV2.1** | 38.5 | 38.3 | 38.9 | 38.9 | 38.6 | 39.0 | 39.0 | 38.9 | 39.5 | 38.6 | 38.6 | 38.2 | 39.2 | 38.8 | 38.3 | 38.0 | 39.0 |  |  |  |  |  |
| **IV2.2** | 39.2 | 38.6 | 38.5 | 38.4 | 38.6 | 38.6 | 38.8 | 38.8 | 38.5 | 38.1 | 38.1 | 38.5 | 38.6 | 38.8 | 38.5 | 38.4 | 38.9 | 39.2 |  |  |  |  |
| **IV2.3** | 38.5 | 38.7 | 38.5 | 38.5 | 38.6 | 38.5 | 38.7 | 38.6 | 38.6 | 38.6 | 38.2 | 38.5 | 38.8 | 38.2 | 38.1 | 38.8 | 38.5 | 38.5 | 38.6 | 38.7 | 38.7 | 38.7 |
| **IV2.4** | 38.7 | 38.8 | 38.6 | 38.7 | 38.9 | 38.9 | 38.7 | 38.5 | 38.7 | 38.4 | 38.6 | 39.0 | 38.8 | 38.7 | 38.6 | 39.0 | 39.0 | 38.9 | 38.8 | 39.0 | 39.0 | 38.8 |
| **IV2.5** | 38.3 | 38.5 | 38.5 | 38.9 | 40.2 | 39.8 | 37.8 | 38.0 | 38.3 | 38.1 | 38.2 | 38.7 | 38.8 | 38.4 | 38.2 | 38.6 | 39.2 | 38.7 |  |  |  |  |
| **IV2.6** | 38.3 | 38.2 | 38.6 | 38.4 | 38.5 | 38.5 | 38.4 | 38.5 | 38.4 | 38.3 | 38.5 | 38.6 | 38.7 | 38.6 | 38.3 | 37.9 | 38.7 | 38.7 | 38.9 | 39.0 | 38.7 | 38.8 |
| **SC.1** | 38.3 | 39.0 | 39.0 | 38.9 | 38.6 | 38.6 | 38.6 | 38.6 | 38.6 | 38.4 | 38.3 | 38.5 | 38.6 | 38.1 | 38.3 | 38.9 | 38.5 | 38.8 | 38.3 | 38.8 | 37.9 | 38.3 |
| **SC.2** | 38.8 | 38.8 | 38.8 | 38.9 | 38.3 | 38.7 | 38.9 | 38.7 | 38.7 | 38.3 | 38.6 | 38.9 | 38.8 | 38.3 | 38.9 | 38.3 | 39.0 |  |  |  |  |  |
| **SC.3** | 39.0 | 37.6 | 39.2 | 38.9 | 38.9 | 38.9 | 38.6 | 38.5 | 38.3 | 38.3 | 38.3 | 38.7 | 38.8 | 38.3 | 38.4 | 38.6 | 38.1 | 38.3 | 38.1 | 38.8 | 38.4 | 38.6 |
| **SC.4** | 38.9 | 38.6 | 39.0 | 38.9 | 39.1 | 38.9 | 38.9 | 38.8 | 39.0 | 38.9 | 38.7 | 39.1 | 38.6 | 38.7 | 39.2 | 38.9 | 38.8 | 39.0 | 38.9 | 39.2 | 38.8 | 38.8 |
| **SC.5** | 38.6 | 38.9 | 39.0 | 38.9 | 38.8 | 38.8 | 38.5 | 38.6 | 38.3 | 38.7 | 38.5 | 38.8 | 38.8 | 38.6 | 39.1 | 38.5 | 39.1 | 38.9 | 38.5 | 39.1 | 38.6 | 38.6 |
| **SC.6** | 39.0 | 38.8 | 39.0 | 38.4 | 38.7 | 39.3 | 39.1 | 38.9 | 38.6 | 38.9 | 38.3 | 38.7 | 38.4 | 38.6 | 38.8 | 38.0 | 39.0 | 38.8 | 38.6 | 39.0 | 38.5 | 38.0 |
| **IV3.1** | 38.4 | 38.8 | 38.5 | 37.8 | 38.3 | 38.5 | 38.5 | 38.7 | 38.2 | 38.3 | 38.5 | 38.5 | 38.6 | 38.2 | 38.0 | 38.7 | 38.6 | 38.2 | 38.5 | 38.1 | 38.7 | 38.6 |
| **IV3.2** | 38.5 | 38.6 | 38.5 | 38.4 | 38.6 | 38.6 | 38.5 | 38.5 | 38.6 | 39.0 | 38.7 | 38.7 | 38.9 | 38.4 | 38.2 | 38.8 | 38.8 | 38.8 | 38.9 | 38.7 | 38.6 | 38.8 |
| **IV3.3** | 40.9 | 38.6 | 38.6 | 38.4 | 38.2 | 38.0 | 38.1 | 38.5 | 38.2 | 38.5 | 38.3 | 38.8 | 38.8 | 38.2 | 38.0 | 39.0 | 38.8 | 38.8 |  |  |  |  |
| **IV3.4** | 38.9 | 38.4 | 38.6 | 38.3 | 38.6 | 38.5 | 38.7 | 38.6 | 39.2 | 38.3 | 38.4 | 38.7 | 38.8 | 38.6 | 38.5 | 39.0 | 39.0 | 38.6 | 38.9 | 38.7 | 38.6 | 38.4 |
| **IV3.5** | 38.6 | 38.4 | 38.4 | 38.3 | 38.6 | 38.6 | 38.7 | 37.9 | 38.5 | 38.7 | 38.3 | 38.8 | 38.2 | 38.2 | 37.9 | 38.9 | 38.6 | 38.5 | 39.0 | 38.9 | 38.9 | 38.8 |
| **IV3.6** | 38.5 | 38.6 | 38.7 | 38.8 | 38.7 | 38.6 | 38.6 | 38.6 | 38.3 | 38.5 | 38.6 | 38.9 | 38.8 | 38.5 | 38.4 | 39.3 | 39.0 | 38.9 | 39.1 | 38.8 | 38.4 | 38.8 |
| **IV4.1** | 38.4 | 39.8 | 39.1 | 38.7 | 38.5 | 38.6 | 38.8 | 39.0 | 38.8 | 38.6 | 38.5 | 38.7 | 38.5 | 38.3 | 39.0 | 38.5 | 38.6 | 39.0 | 38.7 | 38.9 | 38.8 | 39.0 |
| **IV4.2** | 41.0 | 38.2 | 38.4 | 38.5 | 38.5 | 38.1 | 38.6 | 38.1 | 38.2 | 38.7 | 38.1 | 37.6 | 38.6 | 38.0 | 38.6 | 38.0 | 38.8 | 37.9 | 38.0 |  |  |  |
| **IV4.3** | 38.6 | 38.6 | 38.6 | 38.4 | 38.5 | 39.0 | 38.6 | 38.3 | 38.6 | 38.4 | 38.0 | 38.7 | 38.6 | 38.5 | 38.4 | 38.5 | 38.9 | 38.8 | 38.6 | 39.0 | 38.5 | 38.5 |
| **IV4.4** | 38.6 | 38.8 | 38.7 | 39.0 | 38.7 | 38.6 | 39.5 | 38.9 | 38.0 | 38.3 | 38.0 | 38.5 | 38.4 | 37.8 | 37.8 | 38.5 | 38.5 | 38.1 | 37.8 | 38.3 | 37.4 | 37.9 |
| **IV4.5** | 39.0 | 38.3 | 38.7 | 39.1 | 38.8 | 38.1 | 37.9 | 38.3 | 38.4 | 38.0 | 38.2 | 37.8 | 38.7 | 38.1 | 38.7 | 37.7 | 38.8 | 38.5 | 38.1 | 38.7 | 38.0 | 38.3 |
| **Control.1** | 38.8 | 38.5 | 38.7 | 38.6 | 38.1 | 38.8 | 38.6 | 39.0 | 38.3 | 38.1 | 38.3 | 37.7 | 38.5 | 38.2 | 38.6 | 38.8 | 38.8 | 38.8 | 38.2 | 39.2 | 38.0 | 38.5 |
| **Control.2** | 39.0 | 39.0 | 38.8 | 38.9 | 39.1 | 38.6 | 38.4 | 38.8 | 38.4 | 38.7 | 38.5 | 38.7 | 38.7 | 38.3 | 38.1 | 38.4 | 38.5 | 39.1 | 38.3 | 39.3 | 37.9 | 38.7 |
| **Control.3** | 38.8 | 38.5 | 38.5 | 38.2 | 38.1 | 38.8 | 38.7 | 38.8 | 38.3 | 38.2 | 38.3 | 37.8 | 38.6 | 38.3 | 38.1 | 38.2 | 38.5 | 38.8 | 38.0 | 38.6 | 38.0 | 38.6 |
| **Control.4** | 38.0 | 38.4 | 38.3 | 38.6 | 38.3 | 37.6 | 38.1 | 38.5 | 38.3 | 37.8 | 38.5 | 38.7 | 38.5 | 38.2 | 38.7 | 37.9 | 38.1 | 38.6 | 38.0 | 39.0 | 37.4 | 38.4 |

**Additional file 2 Rectal temperature records.**
